# Assessing fuel properties effects of 2,5-dimethylfuran on microscopic and macroscopic characteristics of oxygenated fuel/diesel blends spray

**DOI:** 10.1038/s41598-020-58119-y

**Published:** 2020-01-29

**Authors:** Peng Zhang, Xin Su, Hao Chen, Limin Geng, Xuan Zhao

**Affiliations:** 1 School of Automobile, Chang’an University, Xi’an, 710064 China; 2 State Key Laboratory of Engines, Tianjin University, Tianjin, 300072 China

**Keywords:** Mechanical engineering, Thermoelectric devices and materials

## Abstract

2,5-Dimethylfuran (DMF) is a type of attractive sustainable green energy for diesel engines that is designed to reduce soot emission. This study investigated the effect of fuel properties on the macroscopic and microscopic spray characteristics of four test blends under injection pressures of 90, 120 and 150 MPa and ambient pressure of 5 MPa in a common diesel rail injection system. The macroscopic results indicate that with higher density, lower viscosity and lower latent heating of DMF20, the spray tip penetration and spray area are increased and the average spray angle is slightly increased. Interestingly, the effect of latent heating on the average spray angle is more obvious than that of kinematic viscosity. The microscopic results suggest that higher density, lower viscosity and lower latent heating of DMF20 have an adverse effect on the breakup of small droplets. The results of comparative analysis show that the change rules of the spray parameters remain nearly unchanged with increased injection pressure, and the influence of DMF20 properties produces a different change in different spray parameters with increasing injection pressure. The meaningful conclusion is that the properties of DMF are favourable to improvement of the spray and atomization parameters under high injection pressure.

## Introduction

In the face of serious problems such as energy shortage, energy security, environment pollution and global warming^1^, biofuel has become an increasingly popular and important alternative for engines, especially diesel engines, and offers superior fuel efficiencies, greater power and better durability^2^. In view of lower life-cycle greenhouse-gas emissions, 2nd- and 3rd-generation biofuels are now widely used in diesel engines^3^. Because 2,5-dimethylfuran(DMF) and n-butanol can be produced at large scale through conversion of cellulose, they have shown great promise as sustainable green energy materials^4,5^. Engine experimental studies that investigated the emissions of diesel-DMF blends and diesel-n-butanol blends, indicated that DMF can achieve zero soot emission and that n-butanol can significantly improve soot, CO, and CO_2_ emissions without impacting NOx emissions^6–8^.

Chen *et al*. confirmed that DMF contributes more substantially to lower soot emission than n-butanol^9^. Laser diagnostics and chemical kinetic analysis emphasize that the advantage of DMF lies in its fuel properties instead of its molecular structure^10^. Huang *et al*. noted that blends properties play an important role in engine emissions^11^, as explained by experimental research, which showed that atomization improvement significantly reduces emissions, especially for soot^12^. Among many fuel properties, the cetane number is a highly important factor for combustion and emissions^13^. This conclusion is also supported by Liu *et al*., who reported that cetane number is major factor for soot reduction and is more important than oxygen in fuels and other properties^14^. 2-Ethylhexyl nitrate (EHN) is a cetane number improver that can reduce NOx and soot emissions simultaneously when added to oxygenated fuel-diesel blends, without changing other fuel properties^15,16^. With EHN addition, DMF still significantly reduces soot emissions. Therefore, other fuel properties are also important factors for soot reduction^17^. The physical properties of fuels are key properties in the spray and atomization characteristics, which have an obvious impact on the processes of injection, atomisation, ignition, combustion and emission of engines^18–21^. Although combustion and emission are correlated with key fuel properties, kinematic viscosity affects CO and UHC concentrations^22^. Density has an important relationship with weighted emission^23^. Because droplet lifetime hinges on the activity coefficient in heating and evaporation, the evaporation characteristics of fuel have a certain influence on the combustion process^24–26^. Latent heating reduces soot emissions via longer liquid penetration^27^. In advanced combustion mode, differences in physical properties become increasingly important^28^. Therefore, it is essential to research the influence of fuel properties on the spray process.

In research on spray characteristics, alternative blends for diesel engines improve the atomization characteristics by reducing viscosity and surface tension^29^. However, biodiesel blends show a good spray effect with increased viscosity and density^30,31^. Compared with biodiesel, the spray and atomization effects of dimethyl ether (DME) and n-butanol are significantly better for lower viscosity and surface tension^32–34^. This is also the primary reason for the observation that DME is better than methanol with respect to spray characteristics^35^, and di-n-butyl ether and di-ethyl ether show the same results^36,37^. Oxygenated fuels with different oxygen groups behave with a larger spray angle, shorter spray penetration and smaller droplets than diesel, and the effect depends on the viscosity and property of the oxygenated fuel^38^. Fuel characteristics also obviously affect the microscopic spray characteristics, especially for viscosity and surface tension^39,40^. Compared with viscosity, density plays a more important role for spray, as can be proved by the effect of polyoxymethylene dimethyl ether addition by increasing spray penetration without a significant effect on spray cone angle^41,42^. The comparative study between n-pentanol and n-butanol illustrates that higher density and lower viscosity are instrumental in improvement of spray and atomization characteristics^43^. Gasoline in gasoline-diesel is important to increasing the spray angle and spray area with a slight decrease of the spray penetration distance for better vaporization characteristics^44–47^. Thus far, no research has been conducted on the effect of DMF properties on the spray characteristics. Therefore, it is essential to conduct comparative research on the effect of DMF properties in the spray process.

In this study, four diesel blends were used in a spray measurement system for analysis of optical diagnostic data from a high-speed camera and laser particle size analyser. For the macroscopic angle, the diagnostic data from the high-speed camera were analysed by an image analyser. The macroscopic characteristics (spray tip penetration (STP), average spray angle (ASA) and spray area(SA)) were given and used to quantitatively present the spray quality. For the microscopic angle, the diagnostic data from a laser particle size analyser were statistically analysed. The size and quantity of spray drops (number frequency (NF) and Sauter mean diameter (SMD))were used to characterize the spray breakup and atomization quality. A systematic and full analysis of the latent heating, density, and kinematic viscosity of DMF in the spray process was conducted at three injection pressures. This study is an operational and worthy work for clean energy utilization, selection and control.

## Experimental Setup and Method

### Test system

**Figure 1:**
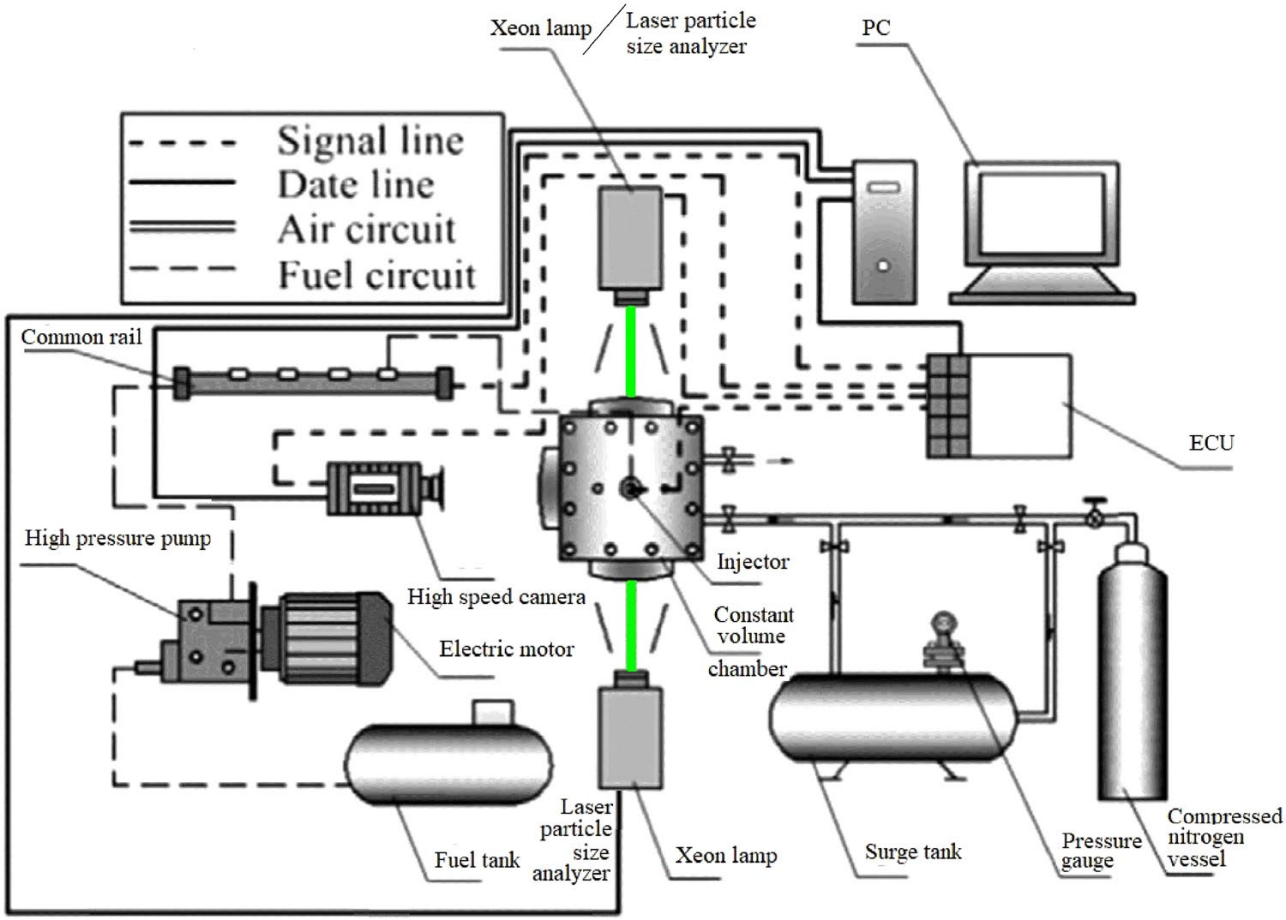
Schematic of macroscopic and microscopic characteristics of the experimental apparatus.

The experimental spray bench is shown in Fig. 1 and consists of a spray system and measurement system. The spray system includes a gas supply unit, a high pressure fuel injection component, a constant volume spray chamber and an electronic control unit (ECU)^48^. In the gas supply unit, pure high-pressure nitrogen was stored in a battle (capacity: 40 L; maximum pressure: 13 MPa). A surge tank was installed between the vessel and the chamber o stabilize the ambient pressure in the chamber. A second pressure valve and an electromagnetic valve were arranged on the outlet of the high-pressure vessel and the inlet of the constant volume chamber, respectively, for supplementary regulation of the ambient pressure. The high pressure fuel injection component is quite similar to the fuel supply system of the diesel engine. The difference is that avariable frequency motor drives and controls a high-pressure pump. Two injectors were connected to a high-pressure rail. One single-hole injector was installed vertically in the head of the constant volume spray chamber for the spray experiments. Another 6-hole injector and a 500 mL measuring cylinder were mounted on a worktop for cleaning of the fuel line. The constant volume spray chamber is a hollow metal cube and three circular observation windows with high-temperature-deformation-resisting quartz glasses and was used to acquire the characteristics of the internal spray. In the real test, no leakage occurred when the ambient pressure of the chamber was 6 MPa. A homebrew was used to control the ECU for regulation and monitoring of the injection parameters (injection pulse, injection times, injection pressure and injection timing). The trigger signals from the ECU controlled the measurement time of the macroscopic and microscopic facilities and the data acquisition time of the software.

The measurement system was divided into macroscopic and microscopic facilities. For the macroscopic measurement, a high-speed camera (FASTCAM-SA7) was used to collect images of the liquid spray configuration and process. Two Xeon lamps supplied the background light necessary to distinguish the fuel from the environment. The particle droplet image analyser (Winner319A) was used to detect local spray microscopic characteristics. A single pulsed Nd:YAG laser with a wavelength of 532 nm and diameter of 10 mm was chosen as the uniform light source for diagnostics. An automatic centring function was applied to ensure that the centre of the transmitter lens and the receiver was consistent. The transmitting end and the receiving end were synchronously moved and positioned at millimetre scales using two electric positioners. The size and quantity of the spray drops were acquired by the laser diffraction method. A PC was used to present and save diagnostic results in real time and to operate the software.

### Tested fuels and test conditions

In this work, diesel was the base oil. Five fuels (DMF, n-butanol, soybean biodiesel, heptane and gasoline) were chosen to compound four test diesel blends. The latent heating, density and kinematic viscosity of the four blends were also measured. The proportion scheme and measured fuel properties are shown in Table 1. The four blends were prepared by blending diesel with 20% fuel mixtures by volume and were named in accordance with the type (acronyms) and concentration (numbers) of blended fuels. As shown in Table 1, the four test blends were named DMF20, BD13B7, NH13G7 and NH16DMF4, respectively.

**Table 1:** Tested fuel composition and fuel properties.

Fuels Vol.% Properties of fuels	DMF20	BD13B7	NH13G7	NH16DMF4
Diesel	80%	80%	80%	80%
DMF	20%	0	0	4%
n-Butanol	0	7%	0	0
Biodiesel	0	13%	0	0
Heptane	0	0	13%	16%
Gasoline	0	0	7%	0
Latent heating (kJ/kg, 20 °C)	**282**.**6**	**282**.**6**	283.8	280
Density (g/L,20 °C)	834	825.8	**800**.**04**	**801**.**68**
Kinematic viscosity (mm^2^ s^−1^, 20 °C)	**4**.**0036**	4.5777	**4**.**0036**	**3**.**994**

To analyse the effects of the spray characteristics for DMF properties, the latent heating of DMF20 and BD13B7 is equal, the kinematic viscosity of DMF20 and NH13G7 is equal, and the density and kinematic viscosity of NH13G7 and NH16DMF4 are highly similar.

The test conditions in this spray experiment are listed in Table 2. The ambient pressure in the chamber was held at 5 MPa, a value quite similar to the compression pressure for a diesel engine when fuel is injected. The injection pressures were 90 MPa, 120 MPa and 150 MPa, and the injection pulse width was set at 2.0 ms. The ambient temperature and the fuel temperature were room temperature.

**Table 2:** Conditions of the experiments.

Injection pressure (MPa)	90, 120, 150
Ambient pressure (MPa)	5
Ambient temperature (K)	293
Fuel temperature (K)	293 (@internal of oil tank)
Nozzle hole diameter (mm)	0.15
Injection times	5
Injection interval (ms)	100

The frame rate of the high-speed camera was set to 10000 fps, and the resolution was set as 896 pixel × 896 pixel. With the adjustment of the imaging distance and manual focusing of the camera lens, the images of the graph paper demonstrated a real size of 896 pixel × 896 pixel is 100 mm × 100 mm. The diameter of the observation windows is 100 mm. The time interval between two neighbouring images is 0.1 ms at a frame rate of 10000 fps. The experiments were repeated 6 times.

### Definition of spray parameters

**Figure 2:**
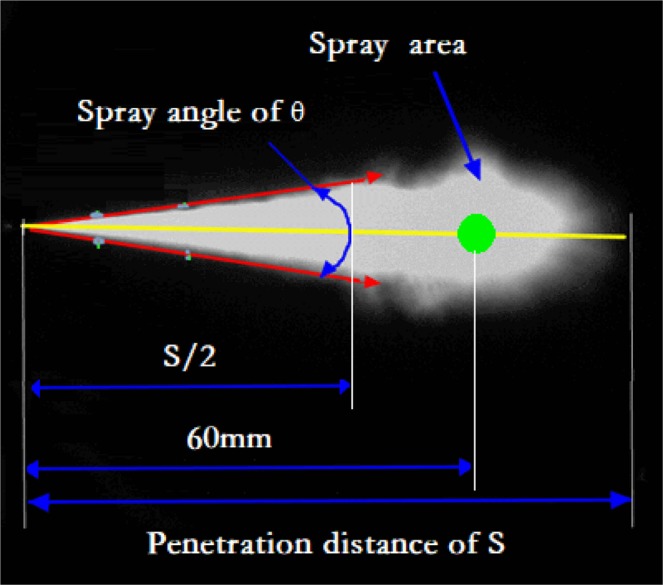
Definitions of spray parameter.

The image analyser in this paper is a self-compiled MATLAB procedure. Using this MATLAB procedure, the macroscopic spray parameters derived from the acquired highspeed images were given for comparative analysis and statistical analysis. These characteristics include STP, spray cone angle and projected SA. The definitions of the abovementioned parameters are shown in Fig. 2. The axial linear distances from the nozzle tip to the farthest spray front is defined as the STP, and the cone angle included between the two lines connecting the nozzle tip and the two periphery points at the half of STP is defined as the spray cone angle. Because the actual edge of the spray is fluctuating and irregular, the triangle and semicircle combination does not reflect the projected SA. Thus, the spray images were converted into grey-scale images for latter binarization with threshold selection, and all spray pixels were directly summed and converted to the projected SA. Therefore, the area of the total pixels covered by the spray contour is defined as the SA.

In addition, the microscopic results are output by the software of the laser particle size analyser. Because of the shortcomings of the laser diffraction method, the size and the quantity of the spray drops in the area of the high-concentration fuel droplets are difficult to accurately measure. In view of the spray image and literature, the measurement positions in this study were set as 60 mm away from the nozzle tip and are marked in Fig. 2.

### Error and uncertainty analysis

The errors and uncertainties originate from the instruments, environment, operating condition, results and analysis method, results and analysis tools and processes, and the changes in the angle of inclination of the fuel spray central axis^46,49^. The errors and uncertainties from the environment and operating conditions could be neglected because the environment and operating conditions in the experiment process were aligned. The errors and uncertainties of the macroscopic spray characteristics originated from the results and analysis methods, tools and processes. The error bars of the macroscopic spray characteristics reflect 30 repeated spray results, and only the averaged results are considered for comparison, discussion and summation.

In addition, the error bars of the macroscopic spray characteristics also contained changes in the angle of inclination of the fuel sprays central axis against the geometrical axis of the nozzle hole^50^. The angle changes depended on cavitation and were affected by fuel properties^49,51^. However, the slight differences in fuel properties have no impact on cavitation. Thus the changes in the angle of inclination of the fuel sprays central axis against the vertical axis were neglected, and the related errors and uncertainties were also neglected.

The errors and uncertainties of the number frequency of drops and the Sauter mean diameter originated from the instruments (Winner319A). For the particle droplet image analyser, the measurement accuracy is ±3% and the repeat error is 3%. The results of the microscopic spray characteristics were taken from the software analyser. The errors and uncertainties were also supplied by the software.

## Experimental Results and Discussion

### The spray of the DMF20

**Figure 3:**
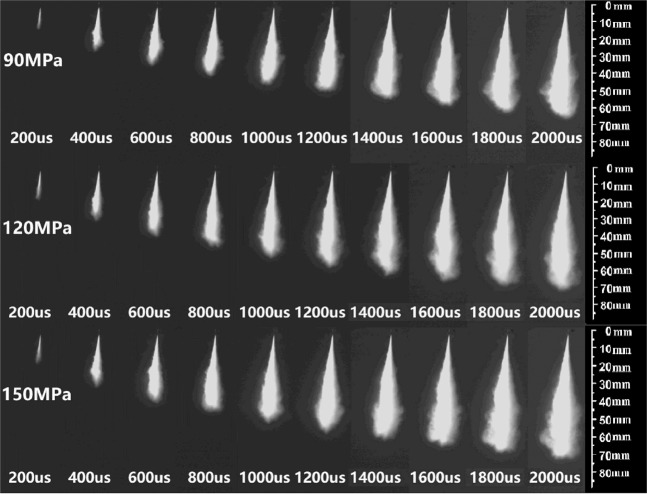
Spray morphology of DMF20 (time in μs) under injection pressures of 90, 120 and 150 MPa.

The spray structure and development process of DMF20 spray is presented in Fig. 3. The vertical length of the spray obviously becomes longer with time. The dark spray image suggests a weakened light scattering effect. More fuel was injected into the constant volume spray chamber with the spray times, and DMF20 sprays display slightly brighter images. This result is interpreted by a strengthened light scattering effect. In Fig. 3, the vertical length of the spray increased with higher injection pressure, which is the result of the momentum and kinetic energy increment of the injected fuel with increased injection pressure^32^.

**Figure 4:**
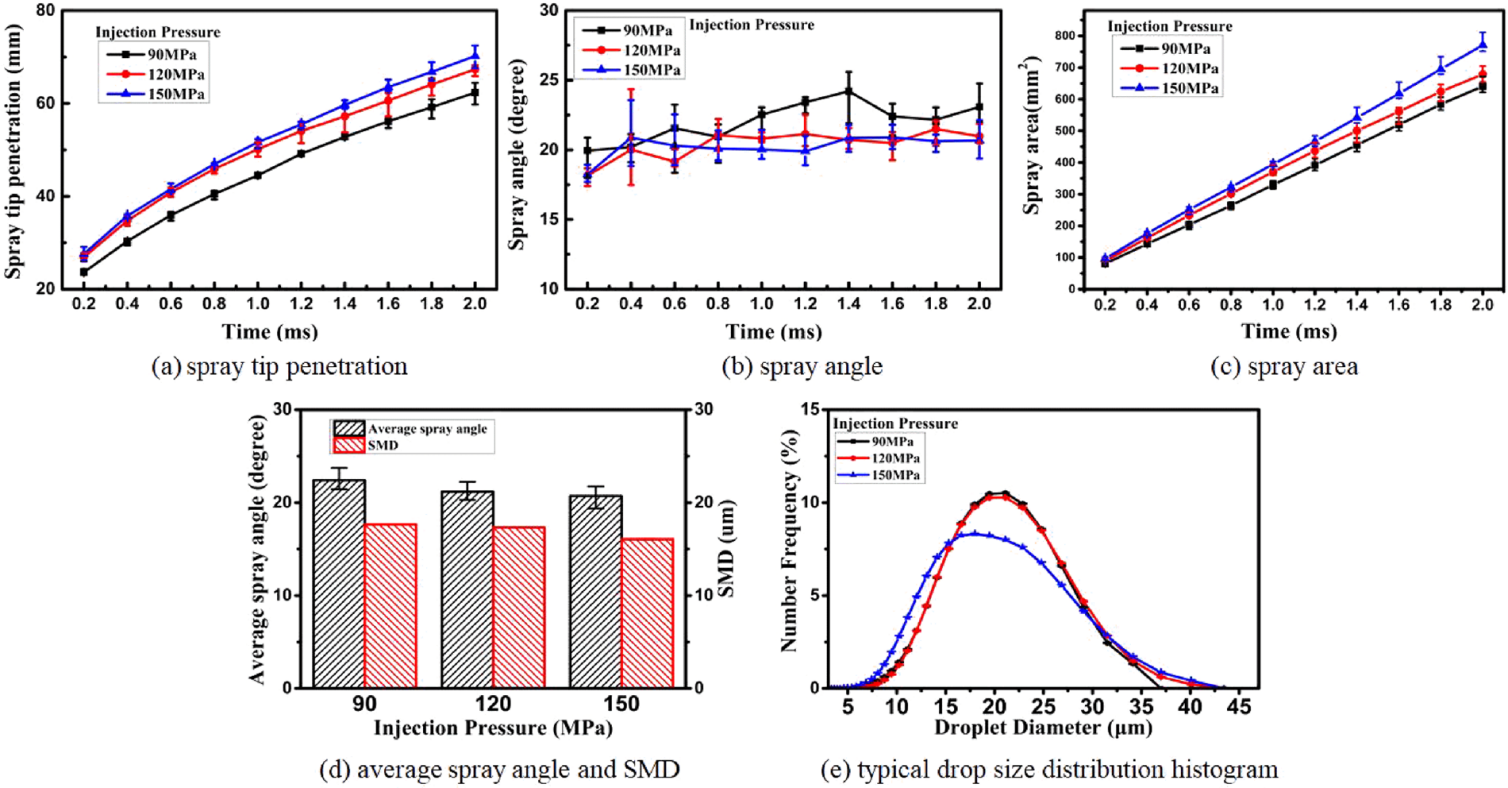
Macroscopic and microscopic spray parameters of DMF20 under different injection pressures (90, 120 and 150 MPa).

The macroscopic results of DMF20 spray under injection pressures of 90, 120 and 150 MPa and 5.0 MPa ambient pressure are revealed in Fig. 4(a–d). Similar to the images in Fig. 3, the STP of DMF20 increases rapidly before 0.6 ms, and the growth trend slows at the later stage. The reasons for this observation are that prior to 0.6 ms, the kinetic energy of the fuel droplet is notably large, and the penetration distance increases rapidly. Subsequently, the high-pressure nitrogen environment and the dissipation of the fuel droplet kinetic energy decrease the velocity of the liquid droplets, resulting in a decrease in the penetration rate. However, the penetration distance continues to increase under the action of gravitational potential energy and residual kinetic energy^52^. As shown in Fig. 4(a,c), a higher injection pressure means higher initial injection momentum, and the STP and SA of DMF20 spray increase with higher injection pressure. This behavior is in accordance with the results of Han *et al*., in which injection pressure is directly proportional to pressure difference^40^. The results in Fig. 4(a) also agree with those of Zhu’s experiments^53^. In consideration of minor changes in spray cone angle with the passage of time, ASA are introduced to appraise the liquid spray here. The average spray cone angle from 1.0 ms to 2.0 ms is defined as the ASA and is shown in Fig. 4(d). The higher initial injection momentum, the weaker nitrogen resistance and the less fuel diffuse along spray normal. Thus, the ASA decreases with increasing injection pressure. Different from the results in Fig. 4(a,c), the injection pressure has less influence on the ASA than on STP and SA. This result is identical with the experimental results of Zhan *et al*.^37^.

The microscopic spray parameters, including SMD and the droplet size distributions of DMF20 under injection pressures of 90, 120 and 150 MPa, are illustrated in Fig. 4(d,e). With higher injection pressure and constant environmental pressure, the differential pressure decreases, and the injected fuel breaks into smaller droplets. Because of fluid viscosity and surface tension, the pressure difference does not have a strong effect on SMD. Nevertheless, SMD decreases with increasing injection pressure in Fig. 4(d). This result is consistent with the result of Han *et al*.^40^. In Fig. 4(e), the range of the DMF20 drops is 5 μm to 45 μm, and the NF of DMF20 reaches a peak value (10.5% and 10.5%, respectively) as the droplet size reaches approximately 21 mm. For the same reason as given for SMD, the peak of the NF curve decreases and moves towards the left with higher injection pressure, and the range of the diameter of the droplet (DOD) is also slightly enlarged. This result demonstrates that high injection pressure is conducive to the breakup of small droplets^40^.

### Effect of fuel properties on the spray of blends

Despite the air drag forces, the internal structure of the injector and the injection parameters, the fuel properties are important to the fuel spray at all times^54^. Based on the introduction, the effect of DMF20 properties on the microscopic and macroscopic spray characteristics are discussed below.

#### Effect of fuel properties on STP

**Figure 5:**
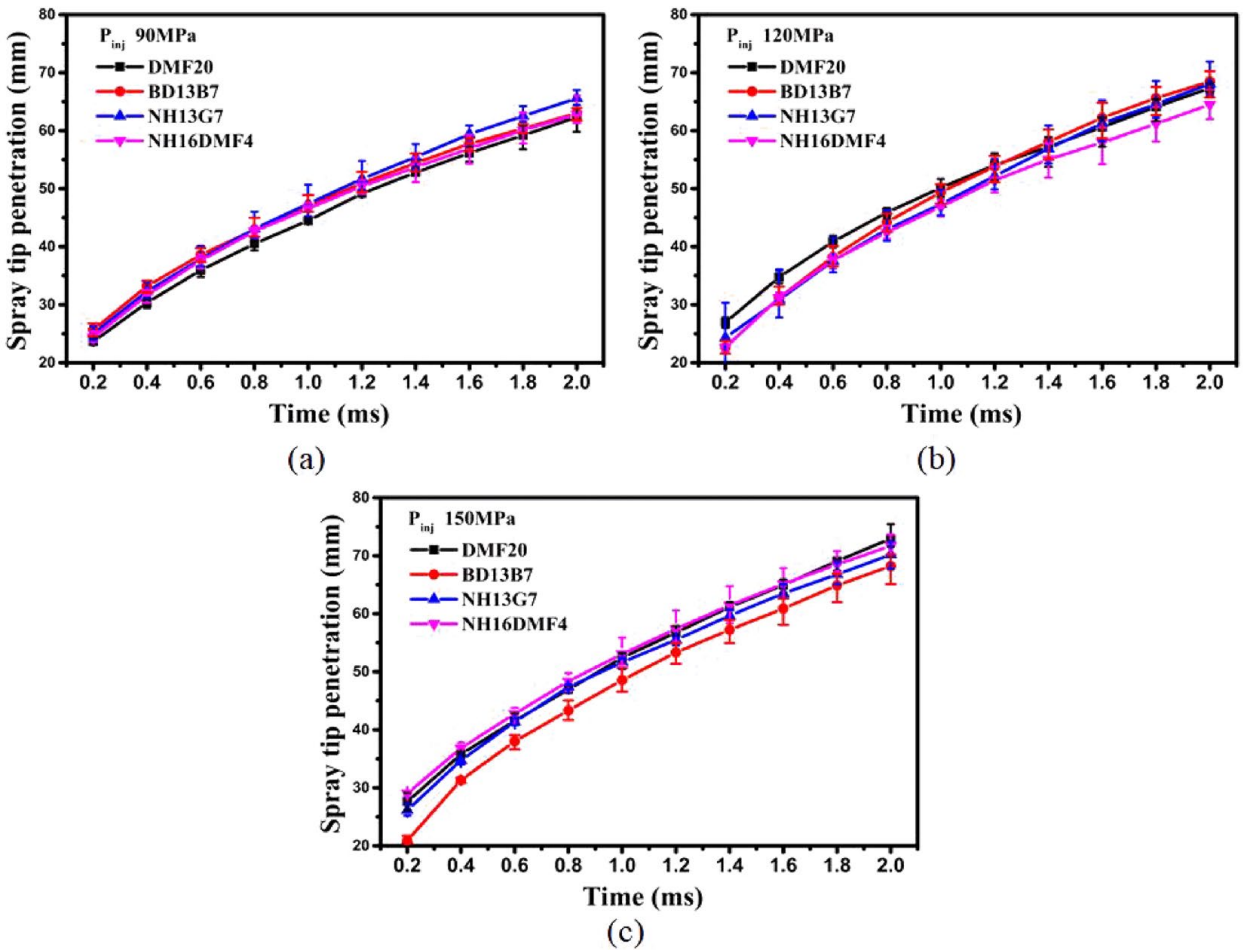
STP of various blends: (**a**) 90 MPa (**b**) 120 MPa (**c**) 150 MPa.

Figure 5 shows the effect of the DMF20 properties on the STP of blends sprayed under various injection pressures. Comparing the STP of DMF20 and BD13B7 under 90 MPa, the STP of BD13B7 was higher than that of DMF20. Two main reasons can be noted for the increase of STP. One reason is that the higher viscosity of BD13B7 increases the droplet size, resulting in decreased momentum of the fuel in spray development, thus leading to greater vertical motion and a longer exercise time of the droplets^55^. The other reason is that the lower density of BD13B7 increases the injection velocity according to analytic turbulent jet theory^56^. Comparing the STP of DMF20 and NH13G7 under 90 MPa, the STP of NH13G7 was higher than that of DMF20 for two main reasons. One reason is that the lower density of NH13G7 increases the injection velocity according to analytic turbulent jet theory^56^. The other reason is that the higher latent heating of NH13G7 increases the difficulty of fuel vaporization. Comparing the STP of DMF20 and NH16DMF4 under 90 MPa, the STP of DMF20 was higher than that of NH16DMF4, and there were two main reasons for the increase of STP. One reason is that the higher density of DMF20 increased the momentum of the droplets. The other reason is that the higher latent heating of DMF20 increases the difficulty of fuel vaporization.

Comparing Fig. 5(a–c), it is interesting to note that the effect of the DMF20 properties on STP changed with increased injection pressure, and the STP of DMF20 was higher than those of the other blends sprayed under 150 MPa. The highest density and lowest viscosity were the two main reasons for the increase of STP. In addition, comparing the STP of NH13G7 and NH16DMF4 under 90, 120 and 150 MPa, the STP of NH13G7 was always higher than that of NH16DMF4. This result further proves that higher latent heating is conducive to higher STP.

#### Effect of fuel properties on ASA

**Figure 6:**
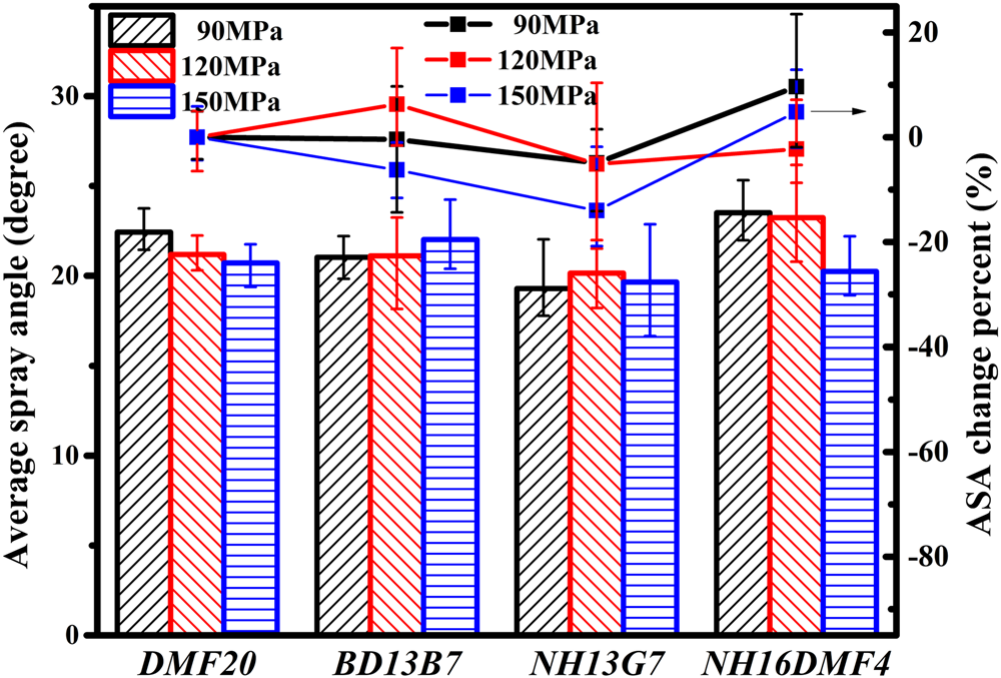
ASA and ASACP of various blends.

The effect of DMF20 properties on the ASA of blends sprayed under various injection pressures is shown in Fig. 6. Because the values of the blends’ ASA were similar, the ASA change percent (ASACP) was introduced for intuitive analysis. The change percent of the blends’ ASA relative to the ASA of DMF20 was defined as the ASACP. Comparing the ASA and ASACP of DMF20 and BD13B7 under 90, 120 and 150 MPa, the SA of DMF20 was higher than that of BD13B7 with lower ASACP. There are also two main reasons for the higher ASA and lower ASACP. One is the same reason as noted for the effect of viscosity in spray breakup. The other reason is that the lower viscosity of DMF20 results in less nitrogen resistance^57^. Comparing the ASA and ASACP of DMF20 and NH13G7 under 90, 120 and 150 MPa, the SA of DMF20 was higher than that of NH13G7, with lower ASACP. For the lower density, the ASA of NH13G7 was lower. Comparing the ASA and ASACP of DMF20 and NH16DMF4 under 90, 120 and 150 MPa, the SA of DMF20 was higher than that of NH16DMF4, with lower ASACP. For the same reason, the ASA of NH16DMF4 was lower.

Comparing the ASA and ASACP of NH13G7 and NH16DMF4, the ASA of NH16DMF4 was higher than that of NH13G7, with lower ASACP because the lower latent heating of NH13G7 increases the vaporization capacity. Interestingly, combined with the previous results, latent heating is a major parameter for ASA. More interestingly, the effect of density and latent heating on ASA was more obvious than that of density and kinematic viscosity.

#### Effect of fuel properties on SA

**Figure 7:**
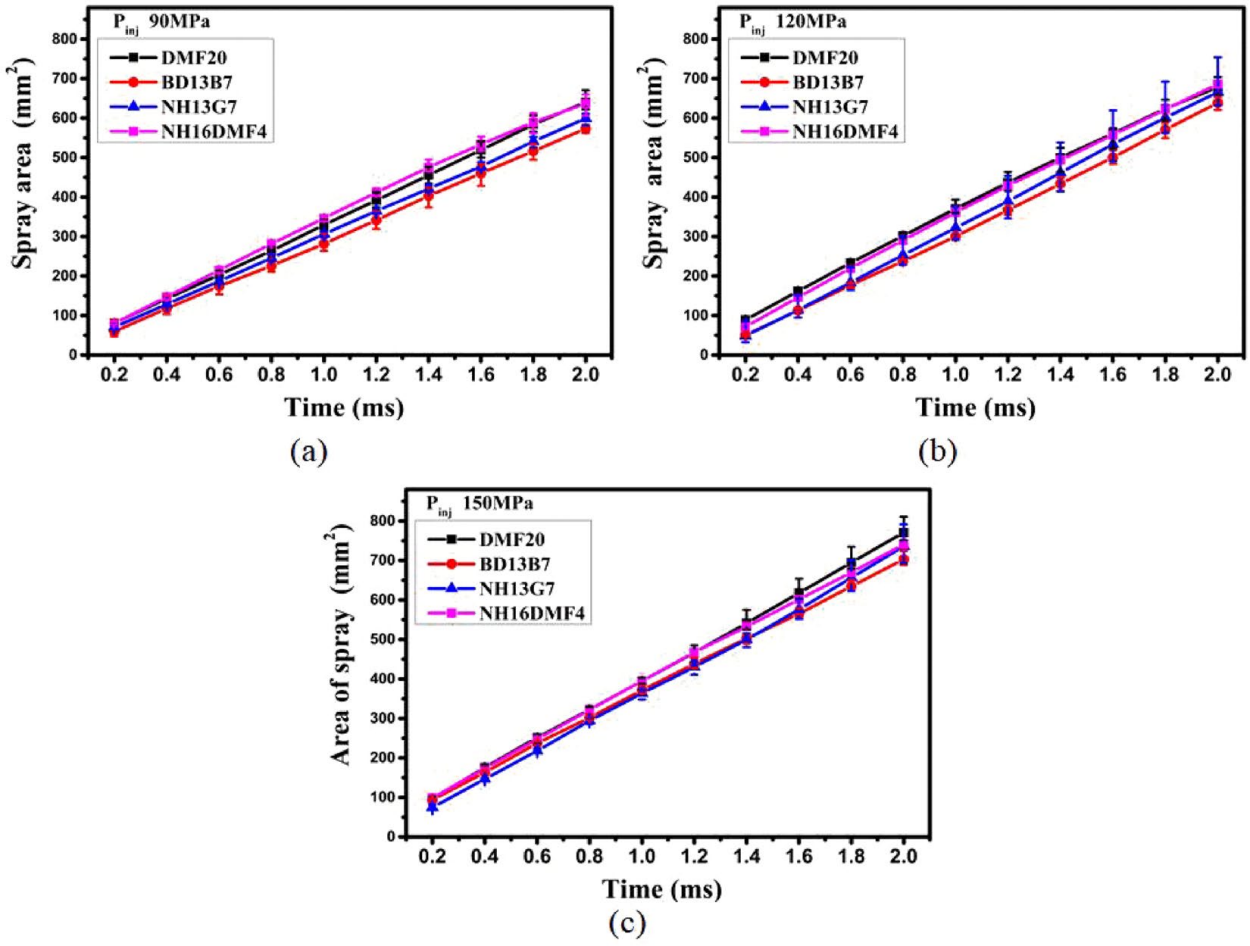
SA of various blends: (**a**) 90 MPa (**b**) 120 MPa (**c**) 150 MPa.

The effect of DMF20 properties on the SA of blends sprayed under various injection pressures is illustrated in Fig. 6. With the guidance of contrast analysis, SA is contributed by STP and ASA. The results show that the effect of STP on SA was stronger than that of ASA. This observation agrees with the conclusions on biodiesel spray^31,40^. Nevertheless, the ASA still affected the results of SA. Instead of STP and ASA, SA might better express the macroscopic spray results. Therefore, under 90 MPa, the DMF20 properties were not propitious to improving the blend spray, but the highest density, lowest viscosity and higher latent heating of DMF20 were advantageous to improving the blend spray under pressures of 150 MPa or higher. Compared with Figs. 5–7, the rules, which are the effect of injection pressure on STP, ASA and SA were in accordance with Fig. 4.

#### Effect of fuel properties on drop size distribution

**Figure 8:**
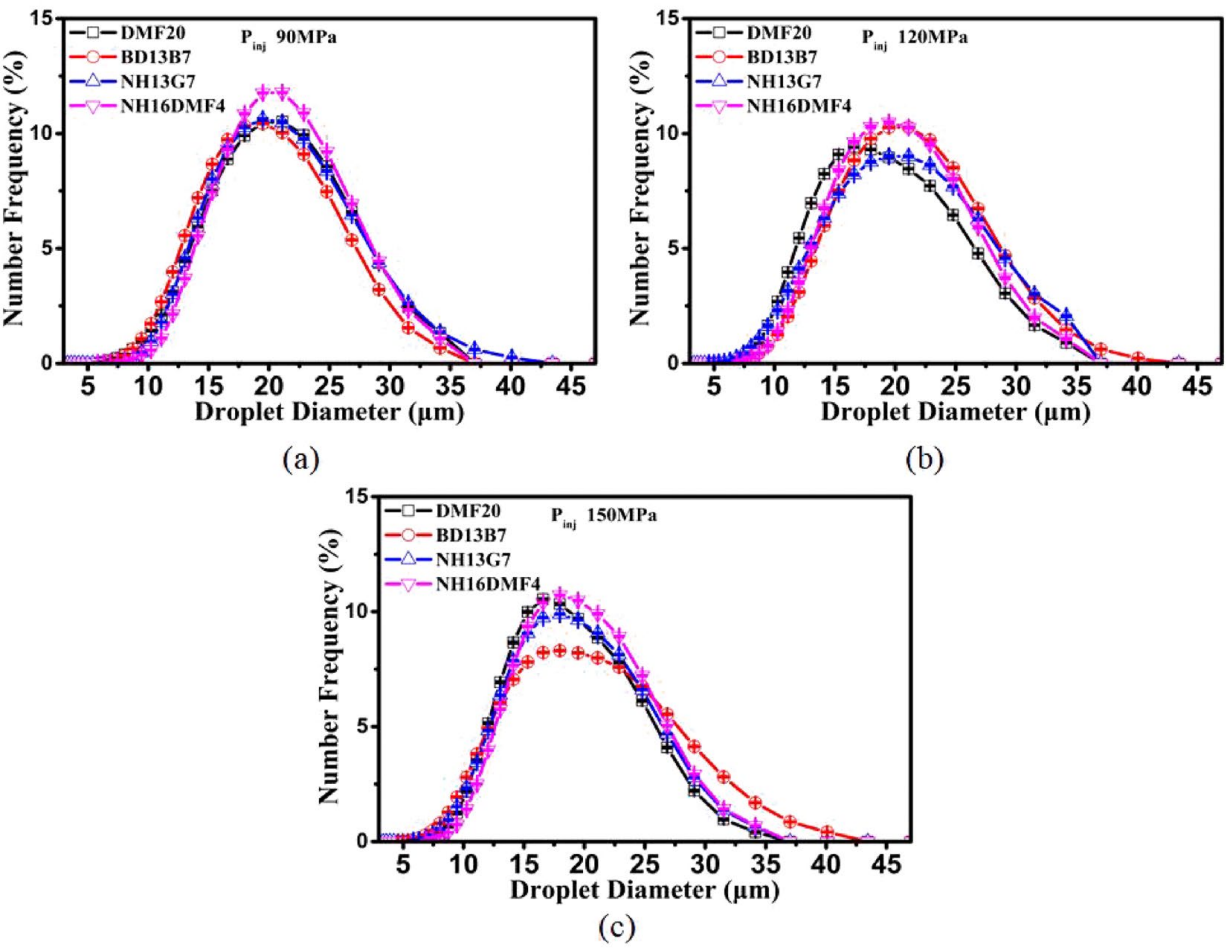
Drop size distribution of various blends: (**a**) 90 MPa (**b**) 120 MPa (**c**) 150 MPa.

Figure 8 illustrates the effect of DMF20 properties on the droplet size distributions of blends atomization under various injection pressure. As shown in Fig. 8(a), the NF values of DMF20 and NH16DMF4 reached peak value (respectively, 10.5% and 11.8%) when DOD was approximately 21 mm. The NF values of BD13B7 and NH13G7 reached peak values (respectively, 10.4% and 10.6%) when the DOD was approximately 19.5 mm. Comparing the curves of DMF20 and BD13B7 in Fig. 8, the curves of BD13B7 moved towards the left, and the peak value of the curves decreased, in opposition to the lower density and higher viscosity. There is one likely reason that the formation of shear splitting is promoted by lower density and higher viscosity. Comparing the experimental results of DMF20 and NH13G7 in Fig. 8, the curves of DMF20 moved towards the right and the peak value of NF increased, in agreement with the higher density and lower latent heating. Comparing the results of DMF20 and NH16DMF4 in Fig. 8, the NF peak value of NH16DMF4 was significantly higher than that of DMF20. Comparing the results of NH13G7 and NH16DMF4, the curves moved towards the right and the peak value of the curves increased, in opposition to the lower latent heating. These results indicate that lower density, higher viscosity and higher latent heating were conducive to the breakup of small droplets.

Comparing the results of the drop size distribution of the four blends under different injection pressures, the curves move towards the left and the peak value of NF decreases with higher injection pressure. The compared results also indicate that the influence of DMF20 properties on the drop size distribution of blends changed with higher injection pressure, and the effect of latent heating on the drop size distribution was weakened by higher injection pressure. The drop size distribution of DMF20 under 150 MPa was the best among the four blends.

#### Effect of fuel properties on SMD

**Figure 9:**
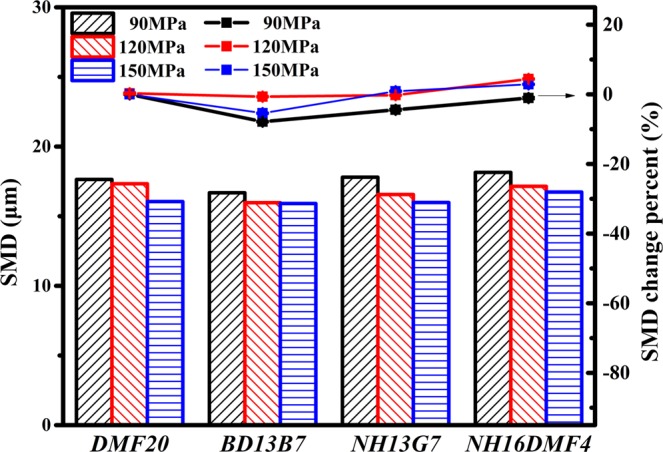
SMD and SMDCP of various blends.

Figure 9 shows the effect of DMF20 properties on the droplet size distributions of the blend atomization under various injection pressures. Because the values of the blends’ SMD were similar, the SMD change percent (SMDCP) was introduced for intuitive analysis. The change percent of the blends’ SMD relative to the SMD of DMF20 was defined as the SMDCP. As illustrated in Fig. 9, the SMD of the four test blends are within the range of 16.686–18.148 μm. The SMD of BD13B7 is the lowest and that of DMF20 is the second lowest. The SMD of NH16DMF4 is the highest among the four test fuels, although Han *et al*. suggested that higher viscosity increases the difficulty of spray breakup, which causes the SMD increase^40^. Ejim *et al*. also noted that viscosity makes the greatest contribution to SMD^58^. These were just results cross to the results in Fig. 9. Luo *et al*. presented the novel idea that the coalescence phenomenon is much stronger with low velocity of the droplets^59^. However, this does not fully explain the results in Fig. 9. According to comparative analysis of the tested fuel properties in Table 1 and the results in Fig. 9, this explanation fays in with the assumed reason for the results of drop size distribution. As shown in Fig. 9, the gaps of SMD and SMDCP among the four test blends decrease with higher injection pressure, a clear demonstration that high injection pressure weakens the effect of DMF20 properties on SMD. The latent heating is the major determinant of SMD under 150 MPa.

## Conclusions

In this study, the effect of DMF properties on the macroscopic and microscopic spray characteristics was investigated under different injection pressure conditions. Four tested blends were used in the study to contrast and analyse the effect of latent heating, density and kinematic viscosity of DMF. The experimental results of STP, ASA, SA, SMD and droplet size distribution are summarized as follows:

1. With the increase in injection pressure, STP and SA increase and ASA and SMD slightly decrease. In addition, the droplet size distribution is improved by increasing the injection pressure.
2. Under 90 MPa, after blending with DMF, STP decreases with lower viscosity, higher density and lower latent heating. ASA slightly increases with lower viscosity and lower latent heating. Interestingly, the effect of latent heating on ASA is more obvious than that of kinematic viscosity. In general, SA, which supplies a better overview of the macroscopic spray result, is more easily affected by STP than SA.
3. Under 90 MPa, with the addition of DMF, the characteristic diameters increase slightly with lower viscosity and higher density, especially for SMD. Unlike other experimental conclusions, the formation of the shear splitting, which is promoted by lower density and higher viscosity, decides the SMD of fuel droplets. The results of the droplet size distribution of the four test blends indicate that higher density, lower viscosity and lower latent heating of DMF have an adverse effect on the breakup of small droplets.
4. The effect rules of DMF20 properties on the macroscopic and microscopic spray parameters changed with the increase in injection pressure. The effect of DMF properties on ASA and SMD was weakened. The effect of DMF properties on STP, SA and droplet size distribution was obviously changed. In general, the properties of DMF were advantageous to improving the macroscopic and microscopic spray characteristics of diesel blends under pressures of 150 MPa or higher.
